# Postvoid Residual Volume After Radical Hysterectomy for Early-Stage Cervical Cancer: Predictive Factors and a Decision-Making Algorithm †

**DOI:** 10.3390/cancers18010024

**Published:** 2025-12-21

**Authors:** Naia Seminario, Vicente Bebia, Ana Luzarraga Aznar, Marta San José, Elvira Vallés, Giulio Bonaldo, Antonio Gil-Moreno, Martina Aida Angeles

**Affiliations:** Department of Gynecological Oncology, Vall d’Hebron University Hospital, Universitat Autònoma de Barcelona, Pg. de la Vall d’Hebron, 119-129, 08035 Barcelona, Spain; naia.seminario@gmail.com (N.S.); ana.luzarraga@vallhebron.cat (A.L.A.); msanjose.hv@gencat.cat (M.S.J.); elvira.valles@vallhebron.cat (E.V.); gulio.bonaldo@vallhebron.cat (G.B.); antonio.gil@vallhebron.cat (A.G.-M.); martina.angeles@vallhebron.cat (M.A.A.)

**Keywords:** hysterectomy, radical, cervix neoplasm, urinary bladder dysfunction, residual urine, postoperative care, postoperative complications

## Abstract

Objective: Our study evaluated the time to normalization of postvoid residual volume after radical hysterectomy and identified risk factors for postoperative bladder dysfunction. We also aimed to establish a predictive threshold for bladder dysfunction on the third postoperative day to develop a decision-making algorithm for postoperative voiding management. Methods: This retrospective, single-center study included early-stage cervical cancer patients undergoing type B1 or C1 radical hysterectomy. Factors associated with elevated postvoid residual volume were analyzed using logistic regression, and the threshold was determined using the Youden index. Results: 67 patients were included: 36 patients (53.7%) underwent C1 radical hysterectomy and 31 (46.3%) B1. At discharge, 13 (19.4%) patients required a catheter: 8 (61.5%) required intermittent catheterization, 5 (38.5%) had a Foley catheter. By postoperative day 3, 49 (73.1%) patients recovered their voiding function. The median time to postvoid residual volume recovery was 1 day (IQR: 1–2) for type B1 and 2.5 days (IQR: 2–5) for type C1 (*p* < 0.01). Compared with B1, C1 radicality was independently associated with a higher risk of postoperative voiding dysfunction (OR = 11.46; 95% CI: 1.75–75.24; *p* < 0.05). Based on these findings, we propose an algorithm for risk-adapted postoperative voiding management: B1 patients can safely have catheters removed on postoperative day 1 without a voiding trial, whereas C1 patients require one. C1 patients with postvoid residual volume ≥170 mL should have delayed catheter removal. Conclusions: Surgical radicality is a risk factor for postoperative bladder dysfunction. In type C1 radical hysterectomy, a postvoid residual volume ≥170 mL on the first postoperative day identifies patients at high risk of delayed recovery, supporting a tailored approach to postoperative voiding management.

## Simple Summary

This retrospective study examined the normalization of bladder function after radical hysterectomy, identifying the predictive factors linked to postoperative bladder dysfunction and defining a postvoid residual volume threshold that could guide postoperative voiding management. A total of 67 patients with early-stage cervical cancer who underwent either type B1 or type C1 radical hysterectomy were recruited. By postoperative day 3, 73.1% of patients had recovered normal voiding. Recovery was faster after B1 surgery than after C1 surgery, with a median recovery of 1 day and 2.5 days, respectively. Type C1 radical hysterectomy was associated with a significantly higher risk of postoperative bladder dysfunction. At discharge, 19.4% of patients still required catheterization. Based on these findings, a risk-adapted management algorithm was proposed: catheters can be safely removed on day 1 after B1 surgery, while C1 patients should undergo a voiding trial, with delayed catheter removal if postvoid residual volume is ≥170 mL.

## 1. Introduction

Radical hysterectomy can be classified into different types based on the extent of tissue removal [1,2,3,4]. The classification proposed by Querleu and Morrow includes types A through D, with varying degrees of radicality and nerve preservation [1,2,3,4]. Type B1 radical hysterectomy, for example, involves section of the paracervix at the level of the urether, with partial resection of the uterosacral and vesicouterine ligaments which allows for the preservation of the neural component of the paracervix [1,2,5,6,7]. C1 radical hysterectomy includes a complete resection of the paracervix at its junction with the internal iliac vessels, the resection of the uterosacral ligament at the rectum, and the vesicouterine ligament at the bladder. The ureter is fully mobilized. In this technique the hypogastric and pelvic splanchnic nerves are visualized and preserved. Type C2 radical hysterectomy involves the same extent of paracervical, uterosacral, and vesicouterine ligament resection as C1, but also includes resection of the neural part of the paracervix below the deep uterine vein, thus damaging the pelvic autonomic nerves [1,2,3,4,5,6,7,8].

Radical hysterectomy is associated with voiding dysfunction [9,10,11,12,13], which is characterized by decreased bladder compliance, increased postvoid residual volume, and reduced activity of the detrusor muscle which can significantly impact patients’ quality of life [11,12,14]. In the early postoperative months (3–6 months), a decrease in bladder capacity and a reduced sensation of urinary urgency have been observed [11,13,14,15,16] while in later stages, complications such as reduced bladder compliance and stress urinary incontinence may occur [11,14,17]. This has been hypothesized to be secondary to the disruption of pelvic autonomic nerves that innervate the bladder, urethral sphincter, and urogenital diaphragm due to loss of bladder neck stability [10,12,13].

Even though nerve-sparing techniques for radical hysterectomy have been shown to improve postoperative bladder function [14,18,19], the risk factors for voiding dysfunction and the management of high postvoid residuals in patients undergoing radical hysterectomy remain poorly defined and heterogeneous [11,13,20]. While it has been described that surgical radicality and prior pregnancies are associated with voiding dysfunction, the impact of the type of surgical approach (open vs. minimally invasive surgery) does not seem to influence outcomes [9]. To our knowledge, no studies have attempted to predict postoperative bladder dysfunction or to identify which patients are at high risk of voiding dysfunction in the early postoperative period. This would avoid unnecessary postoperative catheterization and its associated complications, such as catheter-associated urinary tract infection—one of the most common nosocomial infections—in patients whose bladder function has not yet recovered [21,22,23].

The aim of this study was to evaluate the time to normalize postvoid residual volume in our cohort, and to identify risk factors for high postvoid residual volume. We also aimed to establish a predictive threshold for bladder dysfunction on the third postoperative day that could be identified as early as the first postoperative day and to design a decision-making algorithm for postoperative voiding management.

## 2. Methods

### 2.1. Patients and Study Design

We identified all patients who had a radical hysterectomy for early-stage cervical cancer (IA-IB1) according to FIGO 2018 between 2017 and 2023 at Vall Hebron Barcelona University Hospital. The surgeries were performed by certified gynecologic oncologist and fellows under direct guidance and supervision of an expert. We excluded patients with recurrent cervical cancer, a prior history of urinary disorders, those undergoing radiotherapy and/or chemotherapy, as well as those with missing information regarding postvoid residual volumes. Institutional review board approvals were obtained (PR(AMI)197/2025).

### 2.2. Postoperative Care

According to our institutional protocol, bladder catheterization is removed 24 h after surgery. After the patients’ first spontaneous micturition, the postvoid residual volume is measured using abdominal ultrasound applying to Poston’s formula (Volume = height × width × depth × 0.7) if a sonographer is available, or by catheterization if not [24]. If the postvoid residual volume is <100 mL, the patient is considered to have a normal bladder function. If postvoid residual volume is ≥100 mL on two or more occasions, the catheter is reintroduced overnight, and the protocol is repeated the following day. Patients with bladder void disfunction at discharge are instructed to perform intermittent catheterization or may be discharged with an indwelling catheter according to their preferences and to their ability to perform self-catheterization. Patients are scheduled for an outpatient follow-up visit one week after discharge to reassess postvoid residual volume.

### 2.3. Study Data

Medical databases were carefully examined to collect all relevant information. Patients’ demographic data, tumor type and grade, presurgical FIGO 2018 stage, number of previous pregnancies, type of radical hysterectomy according to Querleu-Morrow classification, surgical approach, date of surgery, postoperative complications according to Clavien-Dindo [25] and date of discharge, and postvoid residual volumes during hospitalization were retrieved from medical records. A lower urinary tract infection was considered present postoperatively when symptoms (dysuria, suprapubic pain, tenesmus, or frequent urination) were accompanied by a positive urine culture. Delayed voiding recovery is defined as >3 days to normalization of postvoid residual volumes.

In accordance with the journal’s guidelines, we will provide our data for independent analysis or for reproducibility of this study in other centers if such is requested.

### 2.4. Statistical Analysis

Data were summarized by median and interquartile rank (IQR) and relative frequencies. Variables were tested for significance using chi-squared test or Fisher test for categorical variables. Univariate and multivariate adjusted logistic regression models were used for the evaluation of the factors associated with a higher postvoid residual volume.

The best predictive threshold of postvoid residual volume has been established using the Youden index.

All statistical tests were two-sided and *p*-values < 0.05 were considered statistically significant. Statistical analyses were conducted using Stata 16.0 (StataCorp, College Station, TX, USA) software.

## 3. Results

A total of 67 women were included in the study. Of these, 49 patients (73.1%) underwent minimally invasive surgery (10 laparoscopy and 39 robotic approach), while the remaining 18 patients (26.9%) underwent laparotomic surgery. Baseline clinical characteristics, preoperative FIGO 2018 staging, and histopathological findings for the entire cohort are summarized in Table 1.

Regarding the extent of radicality, 31 patients (46.3%) underwent type B1 radical hysterectomy, while 36 patients (53.7%) underwent type C1 radical hysterectomy. Postoperative complications were infrequent. Specifically, only four (6.0%) patients developed a lower urinary tract infection, two of them had undergone a type C1 radical hysterectomy and two type B1 radical hysterectomy. Among those affected, just one patient was discharged with an indwelling urinary catheter.

**Figure 1:**
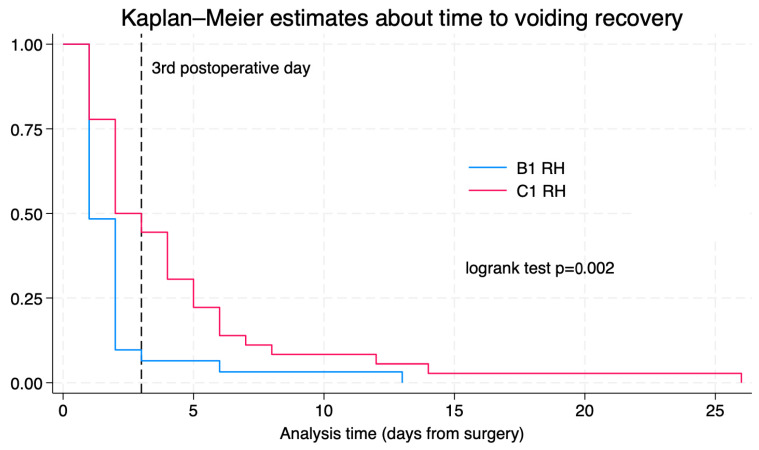
Kaplan–Meier for time to voiding recovery according to surgical radicality.

The median time to recovery of postvoid residual volume was two days (IQR: 1–4) for the overall cohort. When stratified by surgical type, patients who underwent type B1 radical hysterectomy had a median recovery time of 1 day (IQR: 1–2), while those who underwent type C1 radical hysterectomy had a median recovery time of 2.5 days (IQR: 2–5), *p* < 0.01. The median time to discharge was 2 days (range 2–3). At the time of hospital discharge, 13/67 patients (19.4%) required some form of urinary catheterization. Of those, five patients (38.5%) were managed with a Foley catheter, while the remaining eight patients (61.5%) required intermittent catheterization to manage voiding dysfunction. By the third postoperative day, 49 out of 67 patients (73.1%) had recovered voiding function. Among these 49 patients, 29 out of 31 (93.5%) had undergone a type B1 radical hysterectomy, and 20 out of 36 (55.6%) a type C1. As seen in Table 2, in the univariate analysis, type C1 radical hysterectomy was significantly associated with a higher risk of increased postvoid residual volume on postoperative day 1 (OR = 11.6; 95% CI: 2.40–56.12; *p* < 0.01). This association did remain statistically significant in the multivariate analysis after adjusting for potential confounders (OR = 11.5; 95% CI: 1.75–75.24; *p* < 0.05). Figure 1 shows a Kaplan–Meier analysis of postvoid residual volume recovery, along with a log-rank test comparing outcomes between patients who underwent type B1 versus type C1 radical hysterectomy. In contrast, the type of surgical approach—whether minimally invasive or open—was not found to have a significant impact on the development of voiding issues. History of previous pregnancies was not found to be associated with delayed voiding recovery.

**Figure 2:**
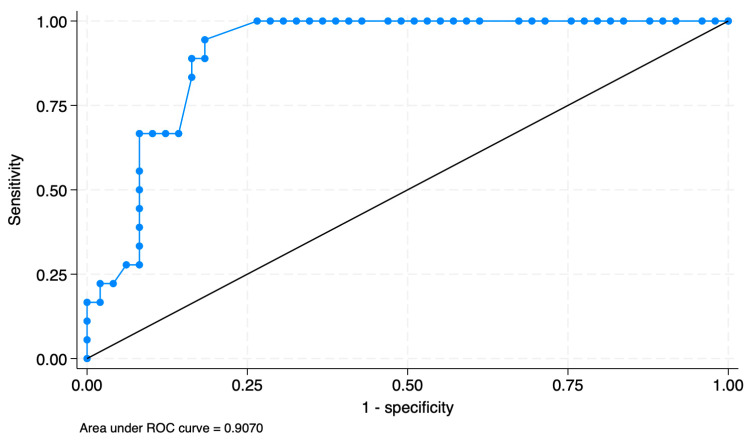
ROC curve analysis of postoperative day 1 post-void residual volume for predicting recovery failure on day 3: optimal cutoff at 167.5 mL.

Additionally, in the C1 radical hysterectomy group, a postvoid residual volume greater than 167.5 mL on the first postoperative day was highly predictive of persistent bladder dysfunction on the third postoperative day. As shown in Figure 2, the ROC analysis demonstrates that a threshold of 167.5 mL on the first postoperative day yields an AUC of 0.907. Of those patients who underwent C1 radical hysterectomy and had a postvoid residual volume > 167.5 mL (17/36) on the first postoperative day, 64.7% (11/17) recovered their voiding function on the 5th day, 76.5% (13/17) on the 7th day, and 94.1% (16/17) on the 14th postoperative day.

## 4. Discussion

It is well established that most patients—up to 72% in some cohorts—undergoing radical hysterectomy experience some degree of voiding dysfunction. The mechanisms underlying this bladder dysfunction are not yet fully understood. Hypothesized causes include injury to sympathetic and parasympathetic nerve fibers, denervation of the detrusor muscle, and damage to the urethral sphincter [12]. Although nerve-sparing techniques have been described, the urinary side effects of radical hysterectomy are still frequent, and there is still no consensus on the timeline for postoperative recovery of voiding function [20,26].

Strategies for urinary catheter withdrawal vary considerably [26,27,28,29]. Early removal—on postoperative day 1—does not appear to increase the risk of urinary retention or catheter reinsertion compared to delayed removal [27]. Several studies have demonstrated that early catheter removal does not result in a higher failure rate during trial of voiding. For example, Shinnick et al. compared trial of voiding failure rates when the urinary catheter was removed before and after discharge. They found that catheterization lasted fewer days in the pre-discharge group (2 ± 2 days) than in the post-discharge group (9 ± 3.5 days, *p* < 0.001). Failure rates of trial of voiding remained comparable between groups (6% vs. 5%, respectively) [28]. Similar outcomes were reported by Mengatto et al.: 25% of patients who had their catheter removed on postoperative day 1 showed a postvoid residual volume greater than 100 mL at their first voiding trial, compared to 34% in the group in those whose catheter was removed on day 7, also without statistically significant differences [29]. Huepenbecker et al. reported that patients who underwent early catheter removal (within 1–5 days) recovered bladder function significantly earlier than those who had delayed removal (>5 days). In their study, the median time to functional recovery was 4 days (IQR: 3–5) in the early group, compared to 8 days (IQR: 7–10) and 13 days (IQR: 11–15) in the intermediate and late removal groups, respectively (*p* < 0.01) [26].

In our cohort, all patients underwent catheter removal 24 h postoperatively; however, 19% were discharged with a urinary catheter. This is comparable to findings by Turnbull et al., who reported that 22% of the patients had a postvoid residual volume >100 mL 48 h after catheter removal and required re-catheterization prior to discharge [30].

Urinary tract infection is a common complication following radical hysterectomy. Identified independent risk factors include catheterization for longer than 7 days and current smoking [21]. In our study, 6.0% of patients developed lower urinary tract infection. This rate is consistent with previous findings by Mengatto et al. (6% in the early catheter removal group) and Turnbull (7%), and notably lower than the 17% reported by Huepenbecker et al. This difference may be explained by the broader urinary tract infection definition used in Huepenbecker’s study, where urinary tract infection was diagnosed based on either symptoms and a positive culture or the administration of antibiotics, even when cultures were negative [26,29,30]. In our study, due to the low number of patients with lower urinary tract infection, we could not evaluate its association with discharge with an indwelling urinary catheter.

In our study, the primary risk factor identified for delayed recovery of normal voiding function was the type of radical hysterectomy performed. Specifically, patients who underwent type C1 procedures had a significantly higher risk of developing voiding dysfunction compared to those who underwent type B1 surgeries. Interestingly, this finding contrasts with the results reported by Zapardiel et al., who observed that both type B1 and type C2 radicality were associated with a higher risk of delayed voiding recovery than C1—with type C2 conferring the greatest risk [9]. Also, in our study, previous pregnancies were not significantly associated with delayed voiding recovery. The discrepancy with findings from Zapardiel et al. could be attributed to various factors: on the one hand, their definition of delayed voiding recovery as more than seven days. On the other hand, a recent anatomical study has shown that bladder innervation from the pelvic splanchnic nerves follows three distinct courses, with only a proportion of these branches being preserved depending on the extent of radicality and on specific technical steps, particularly the management of the posterior layer of the vesicouterine ligament. In nerve-sparing procedures, type C1 radical hysterectomy is estimated to preserve a greater proportion of bladder nerve branches than type C2, whereas type B procedures generally avoid dissection close to the deep autonomic nerves adjacent to the deep fascial plane, thereby minimizing the risk of nerve injury and therefore minimizing the postoperative bladder dysfunction [31]. This reasoning supports the increased risk of postoperative voiding dysfunction of type C1. The explanation for the heterogeneous bladder dysfunction outcomes in type B radical hysterectomy reported across studies, including the findings of Zapardiel et al., might be, therefore, the variability in nerve exposure and identification across surgeons and centers, that may cause some nerves to become inadvertently damaged.

Finally, our results are consistent with theirs regarding the surgical approach, as no significant difference was found between open and minimally invasive procedures in the likelihood of delayed voiding function recovery.

**Figure 3:**
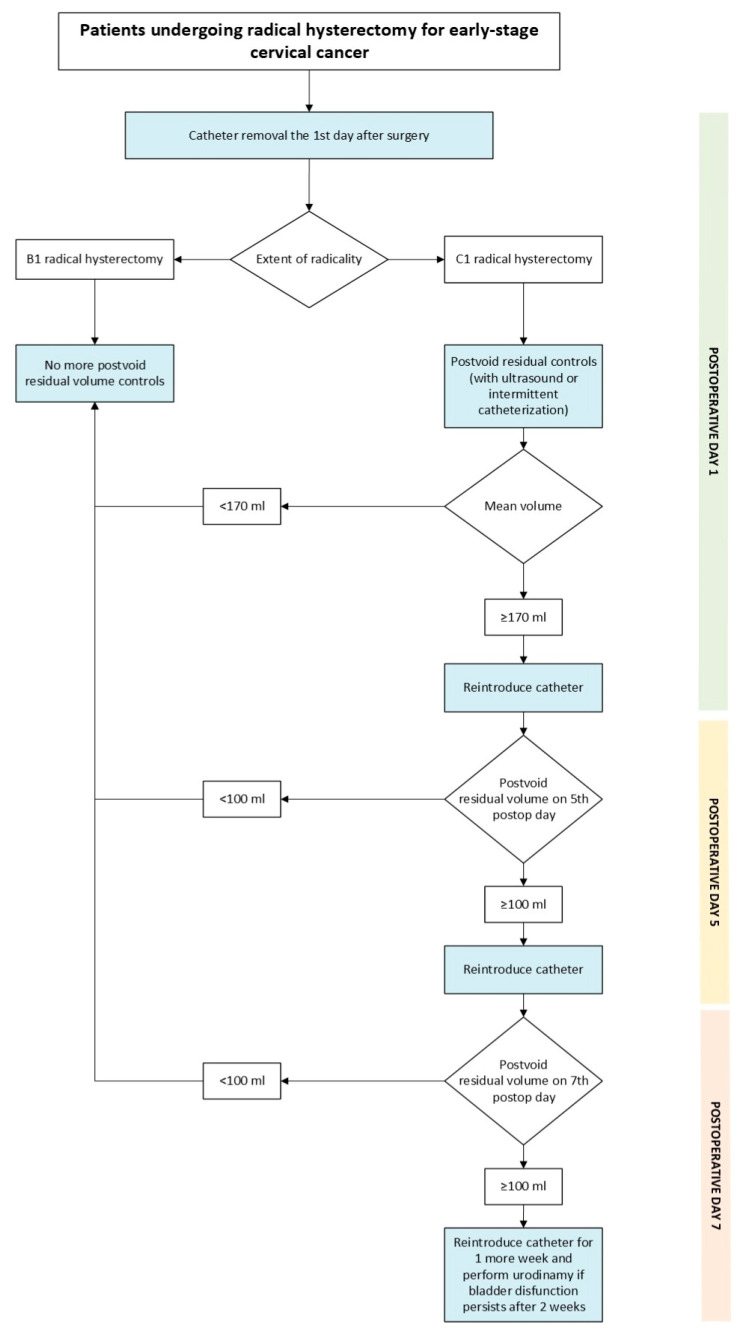
Proposed algorithm.

The lack of standardized criteria for defining voiding dysfunction remains a significant challenge in both clinical practice and research. Establishing a clear postvoid residual volume threshold is essential to ensure consistency in patient follow-up and management. To date, the literature does not provide a validated cut-off value to guide clinical decision-making. In our study, we identified, among patients who underwent C1 radical hysterectomy, a postvoid residual volume threshold of 167.5 mL on the first postoperative day, which can reliably predict those at risk of developing voiding dysfunction (>100 mL at third day). For practical purposes, a threshold of 170 mL will be applied to simplify clinical management. This threshold could not be calculated for the B1 group because 95% of patients recovered their voiding function on the first postoperative day. Based on these findings, we propose the algorithm shown in Figure 3 to guide postoperative care. Patients undergoing B1 radical hysterectomy are considered at low risk for high postvoid residual volumes and therefore do not require postvoid residual volume assessment following catheter removal on the first postoperative day. Conversely, patients undergoing a C1 procedure should be stratified according to the postvoid residual volume measured on the first postoperative day, using a threshold of 170 mL. Patients with a residual volume below this threshold are classified as low risk, and the catheter can be removed and no more tests will be needed. Patients with a residual volume equal to or above 170 mL are considered high risk, in which case the catheter should be reintroduced and no further testing is performed until postoperative day three.

The scope is to personalize treatment—minimizing urinary tract manipulation that can increase urinary tract infections in low-risk patients and in patients whose bladder function has not yet recovered—while offering a standardized approach that ensures consistent management for all patients.

To our knowledge, this is the first study to propose a method for predicting voiding dysfunction after radical hysterectomy. We applied a quantitative definition to measure postvoid residual volume. Additionally, it is one of the few studies that analyze the risk factors associated with this postoperative complication. The urinary catheter was removed at the same postoperative time point for all patients, ensuring homogeneity in patient care.

However, our study has several limitations. It is retrospective in nature and was conducted at a single institution, which may limit the external validity of the findings. Additionally, while we focused on early postoperative voiding dysfunction as a primary complication, we did not include long-term follow-up data to assess the evolution or resolution of the dysfunction over time, which should be evaluated in future studies.

With growing evidence supporting the benefits of early urinary catheter removal, it is now essential to personalize the management of voiding dysfunction in patients undergoing radical hysterectomy. This involves identifying both high- and low-risk patients based on the evaluated risk factors and providing different postoperative care to minimize urinary tract manipulation.

We believe that future prospective, multicenter studies are needed to validate our findings and provide more robust data to validate the decision-making algorithm proposed.

## 5. Conclusions

Surgical radicality strongly influences bladder recovery after hysterectomy with type B1 showing low risk and safe catheter removal on the first postoperative day without voiding trial whereas type C1 requires a voiding trial on day one. C1 patients can be stratified by a 170 mL residual volume threshold into low risk allowing catheter removal and high risk requiring reintroduction and delayed testing. This risk adapted strategy minimizes unnecessary urinary tract manipulation and enhances postoperative care.

## Figures and Tables

**Table 1:** Baseline characteristics, surgical radicality and approach, FIGO stage, intraoperative and postoperative data, and histology.

Variable [Mean (SD), Median (IQR) or *n* (%)]	Total (*n* = 67)
BMI (kg/m^2^)	23.4 (21.4–29.7)
Approach	
Laparotomy	18 (26.9)
Laparoscopy	10 (14.9)
Robotic-assisted	39 (58.2)
Type of radicality	
B1	31 (46.3)
C1	36 (53.7)
Preoperative FIGO stage (2018)	
IA1	1 (1.5)
IB1	48 (71.6)
IB2	14 (20.9)
IIA1	4 (6.0)
Operative time	270 (240–290)
Estimated blood loss	100 (50–250)
Length of stay	2 (2–3)
Intraoperative complications	3 (4.5)
Postoperative complications	7
Clavien-Dindo	
II	6
III *	1
Type of postoperative complications	
Gastrointestinal	1
Wound dehiscence	1
Urinary Tract Infection	4
Postoperative bleeding	1
Readmission	1 (1.5)
Reintervention	1 (1.5)
Histology	
Squamous	36 (53.7)
Adenocarcinoma HPV-related	28 (41.8)
Adenocarcinoma HPV-unrelated	3 (4.5)

* III postoperative complication was dehiscence of the cupola that required reintervention.

**Table 2:** Univariate and multivariate regression analysis of factors associated with postvoid residual volume on the first postoperative day.

Variable	Univariate Analysis	*p* Value	Multivariate Analysis	*p* Value
Mean post-void residual urine (1st day)	1.01 (1.01–1.02)	<0.001	1.01 (1.01–1.02)	0.001
Radicality				
B1 RH	Ref			
C1	11.60 (2.40–56.12)	0.002	11.46 (1.75–75.24)	0.011
Approach				
Laparotomy	Ref			
Laparoscopy	0.86 (0.16–4.55)	0.856		
Robotic-assisted	0.60 (0.18–2.05)	0.416		
Parity				
Nulliparous	ref			
One previous pregnancy	0.6 (0.08–4.44)	0.617		
>1 previous pregnancy	1.4 (0.33–6.24)	0.623		

## Data Availability

The data presented in this study are available on request from the corresponding author due to privacy reasons.
